# Self-organized lateral inhibition improves odor classification in an olfaction-inspired network

**DOI:** 10.1186/1471-2202-14-S1-O12

**Published:** 2013-07-08

**Authors:** Bahadir Kasap, Michael Schmuker

**Affiliations:** 1 Theoretical Neuroscience, Institute of Biology, Freie Universität Berlin, Berlin, 14195, Germany; 2 Bernstein Center for Computational Neuroscience Berlin, Berlin, 10115, Germany

## 

The insect olfactory system is capable of classifying odorants by encoding and processing the neural representations of chemical stimuli. Odors are transformed into a neuronal representation by a number of receptor classes, each of which encodes a certain combination of chemical features. Those representations resemble a multivariate representation of the stimulus space [1]. The insect olfactory system thus provides an efficient basis for bio-inspired computational methods to process and classify multivariate data.

Olfactory receptors typically have broad receptive fields, and the odor spectra of individual receptor classes overlap. From the viewpoint of multivariate data processing, overlapping receptive fields cause correlation between input variables (*channel correlation*). In previous work, we demonstrated how lateral inhibition in an olfaction-inspired network reduced channel correlation [2,3]. Decorrelation was achieved by setting the strength of lateral inhibition between two channels according to their correlation, which we pre-computed from the input data.

Here, we propose unsupervised learning of the lateral inhibition structure. The lateral inhibition synapses support inhibitory spike-timing dependent plasticity (iSTDP) [4,5]. After exposing the network to a sufficient number of input samples, the inhibitory connectivity self-organizes to reflect the correlation between input channels. We show that this biologically realistic, local learning rule produces an inhibitory connectivity that effectively reduces channel correlation and yields superior network performance in a multivariate scent recognition scenario.
